# Acute neck pain caused by septic arthritis of the lateral atlantoaxial joint with subluxation: a case report

**DOI:** 10.1186/s13256-015-0651-3

**Published:** 2015-08-15

**Authors:** Takashi Kobayashi, Naohisa Miyakoshi, Eiji Abe, Toshiki Abe, Kazuma Kikuchi, Yoichi Shimada

**Affiliations:** 1 Department of Orthopedic Surgery, Akita Kousei Medical Center, 1-1-1 Iijima-Nishifukuro, Akita, 011-0948 Japan; 2 Department of Orthopedic Surgery, Akita University Graduate School of Medicine, 1-1-1 Hondo, Akita, 010-8543 Japan

**Keywords:** Cervical spine, Lateral atlantoaxial joint, Septic arthritis, Surgical immobilization, Vertebral osteomyelitis

## Abstract

**Introduction:**

Crystal-induced arthritis of the lateral atlantoaxial joint may be intimately involved in acute neck pain in the elderly. Patients typically have a good prognosis, and symptoms usually subside within a few weeks. On the other hand, septic arthritis of the lateral atlantoaxial joint requires early diagnosis and antibiotic treatment. Diagnostic delay is a risk factor for an unfavorable outcome of vertebral osteomyelitis. Even though septic arthritis of the lateral atlantoaxial joint is a very rare clinical entity, it is important to differentiate septic arthritis from crystal-induced arthritis.

**Case presentation:**

A 53-year-old Japanese man presented with neck pain, stiffness, and loss of power of his left upper extremity which started 20 days before his visit to our hospital. A physical examination revealed a limited range of motion of his neck, with rotation being especially very restricted. Atlantoaxial subluxation was seen on plain radiography of his cervical spine. During puncture of the lateral atlantoaxial joint, clear yellow fluid was collected. Cultures later grew methicillin*-*sensitive *Staphylococcus aureus*. He was diagnosed with septic arthritis of the lateral atlantoaxial joint with atlantoaxial subluxation. After diagnosis, intravenous administration of antibiotics was begun. The atlantoaxial region was stabilized with the Brooks procedure. Plain radiography showed complete bone union 8 months after operation. At a follow-up evaluation 7 years after initial onset, he had complete relief of neck pain, and there were no neurological abnormalities.

**Conclusions:**

A patient with septic arthritis of the lateral atlantoaxial joint with subluxation presenting with acute neck pain was successfully treated with antibiotics and fusion surgery. In patients with persistent neck pain, septic arthritis of the lateral atlantoaxial joint should be considered and further examinations performed.

## Introduction

Crystal-induced arthritis of the lateral atlantoaxial joint may be intimately involved in acute neck pain in the elderly [1]. Patients typically have a good prognosis, and symptoms usually subside within a few weeks. On the other hand, septic arthritis of the lateral atlantoaxial joint requires early diagnosis and antibiotic treatment [2–5]. Diagnostic delay is a risk factor for an unfavorable outcome of vertebral osteomyelitis [6]. Even though septic arthritis of the lateral atlantoaxial joint is a very rare clinical entity, it is important to differentiate septic arthritis from crystal-induced arthritis.

Atlantoaxial subluxation associated with infection at the pharynx and its surrounding tissues is called Grisel’s syndrome [7]. Grisel’s syndrome has also been described in association with postoperative inflammation in surgical conditions such as tonsillectomy and adenoidectomy, in which a clear infective factor is not always proved [8, 9]. The majority of reported cases occurred in patients under 21 years of age [10]; it is rare in adults [11].

The purpose of this paper is to report an extremely uncommon case of septic arthritis of the lateral atlantoaxial joint with subluxation, along with its clinical and imaging features.

## Case presentation

A 53-year-old Japanese man presented with neck pain, stiffness, and discomfort of his left upper extremity which started 20 days before his visit to our hospital. He was referred to our department for detailed examination of prolonged neck pain. At initial onset of his neck pain he had high fever. He had not been exposed to tuberculosis and had no history of recent head or neck injuries or diabetes mellitus.

**Fig. 1:**
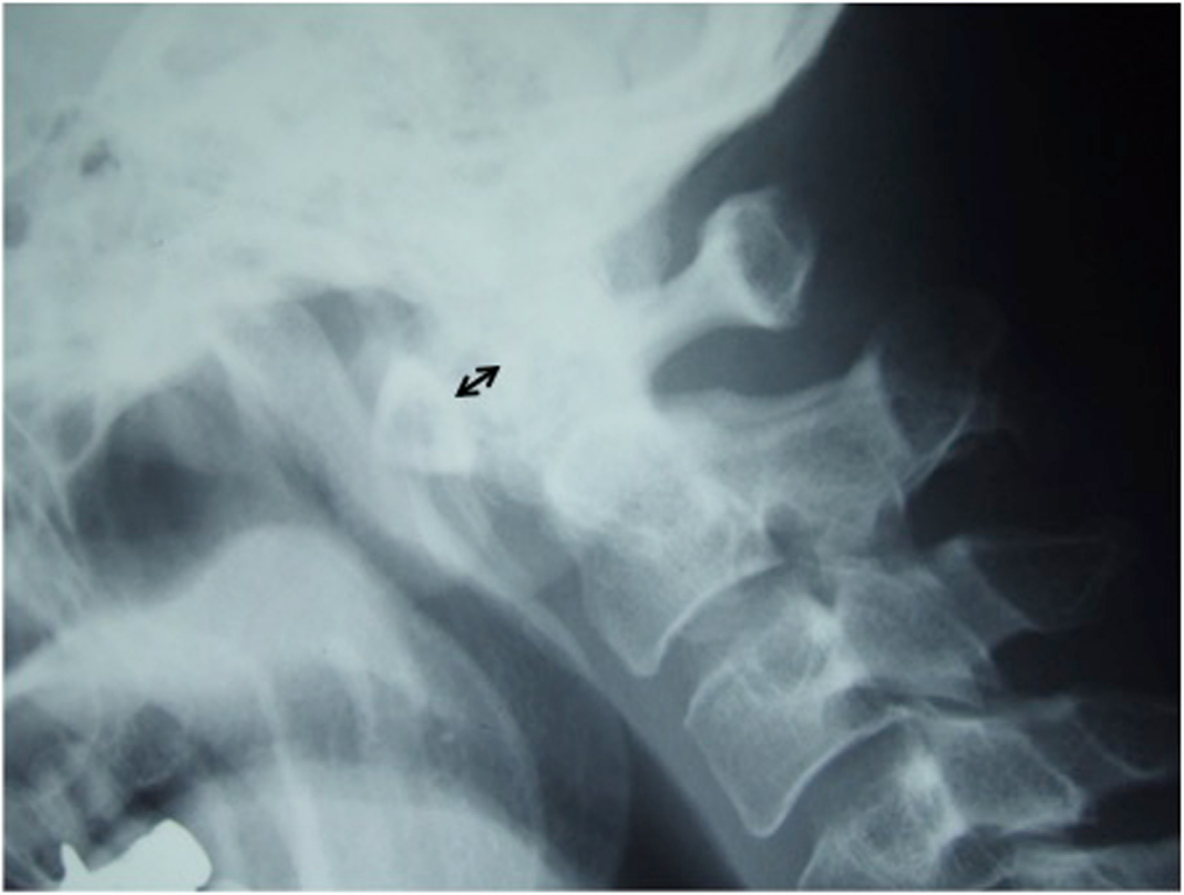
Plain lateral radiograph on admission. The atlantoaxial distance is 7mm (*bidirectional arrow*)

**Fig. 2:**
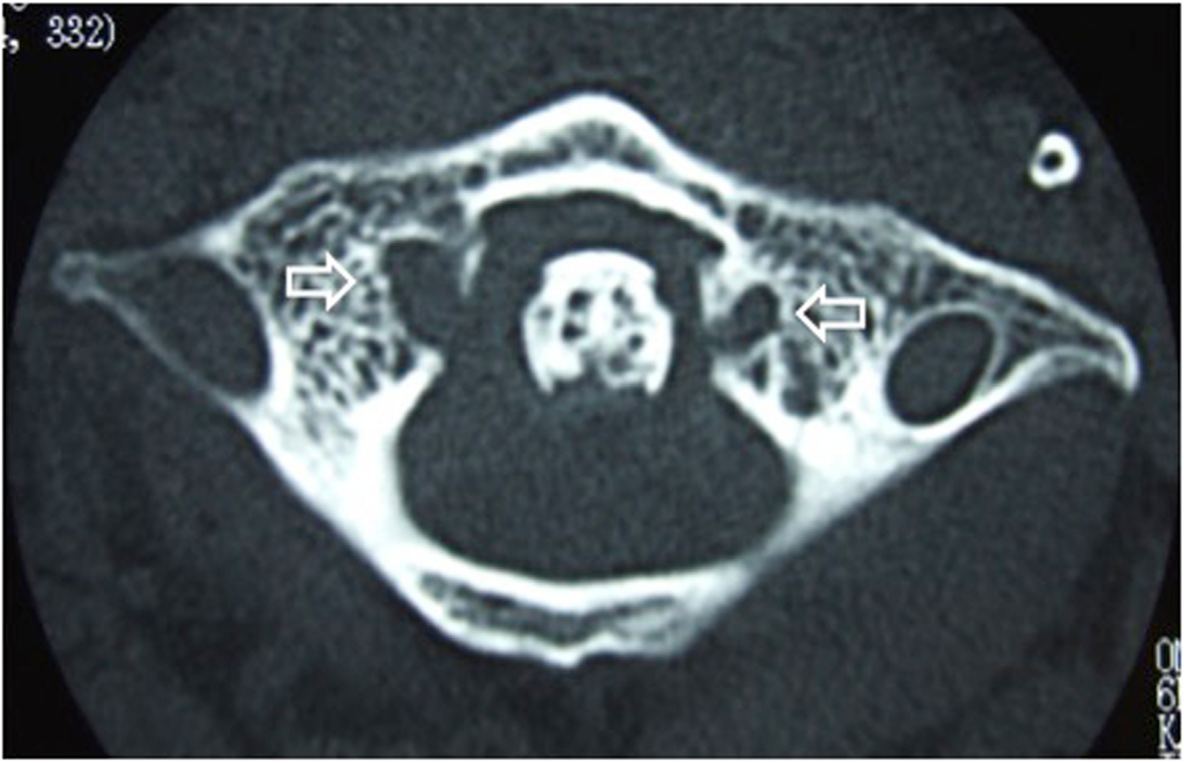
Computed tomography on admission. Erosive changes of the bilateral lateral masses of the atlas (*open arrows*) are visible

**Fig. 3:**
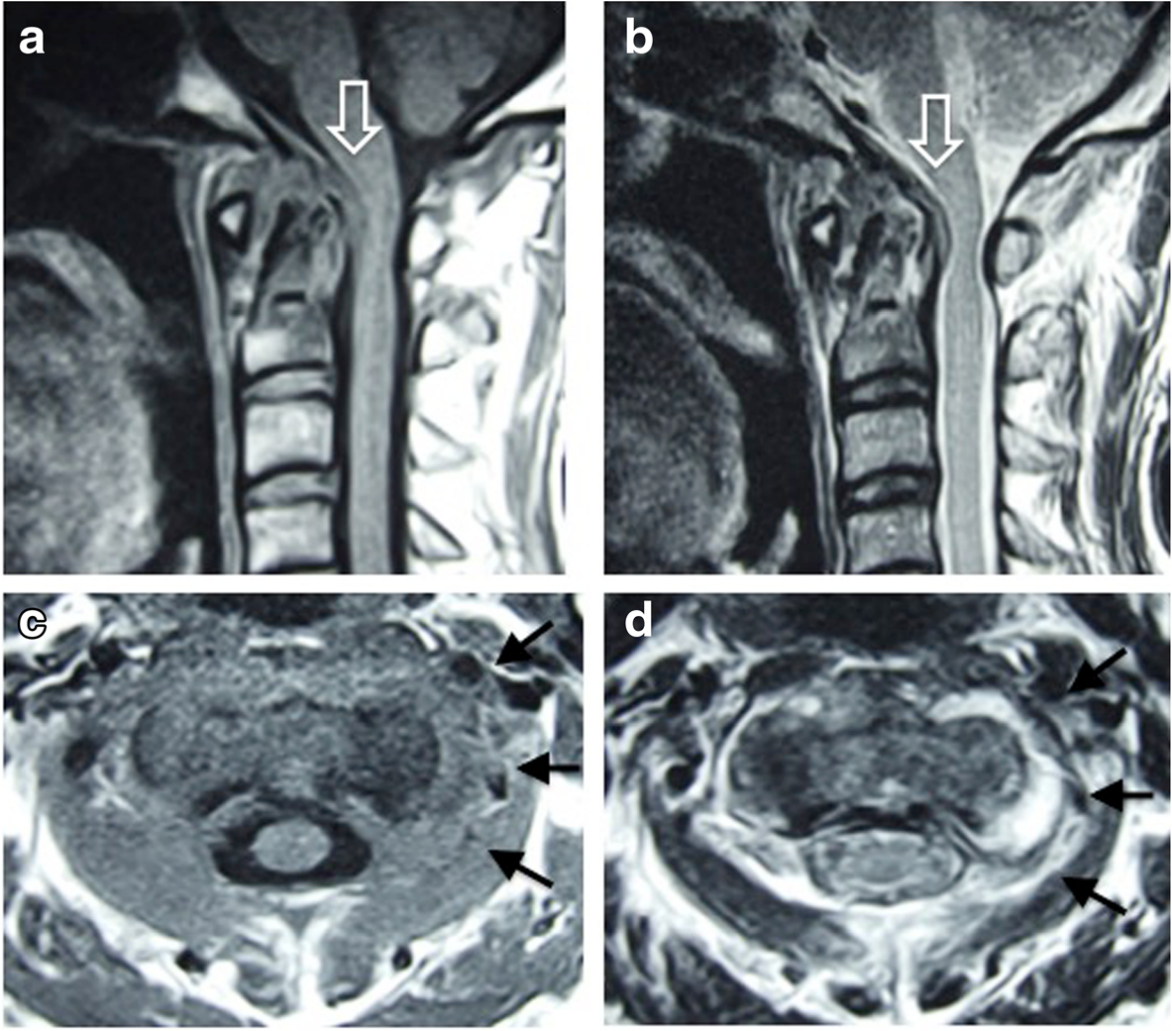
Magnetic resonance imaging on admission. Sagittal imaging (**a**, **b**) shows cord compression due to a pseudotumor around the dens (*open arrow*). Axial imaging shows heterogeneously low signal intensity around the left lateral atlantoaxial joint on T1-weighted imaging (**c**, *arrows*) and high signal intensity on T2-weighted imaging (**d**, *arrows*)

A physical examination revealed a limited range of motion of his neck, with rotation being especially very restricted. His motor strength and sensory functioning of upper and lower extremities were unremarkable, but he had hyperreflexia of biceps tendon reflex, triceps tendon reflex, patellar tendon reflex and Achilles tendon reflex. Atlantoaxial subluxation was seen on plain radiography of his cervical spine (Fig. 1). Computed tomography (CT) showed erosive changes of the bilateral lateral masses of the atlas (Fig. 2). Sagittal magnetic resonance imaging (MRI) studies showed cord compression due to a mass around the dens (Fig. 3a, b). Axial MRI studies showed heterogeneously low signal intensity around the left lateral atlantoaxial joint on T1-weighted imaging (Fig. 3c) and high signal intensity on T2-weighted imaging (Fig. 3d).

He was admitted to our hospital 6 weeks after onset of symptoms because his severe neck pain continued. Laboratory examinations at admission showed a white blood cell count of 8200 per mm^3^ (normal range, 3500 to 9300 per mm^3^), C-reactive protein of 2.0mg/dL (normal range, 0 to 0.3mg/dL), and an erythrocyte sedimentation rate of 42mm/hour (normal range, 2 to 10mm/hour).

**Fig. 4:**
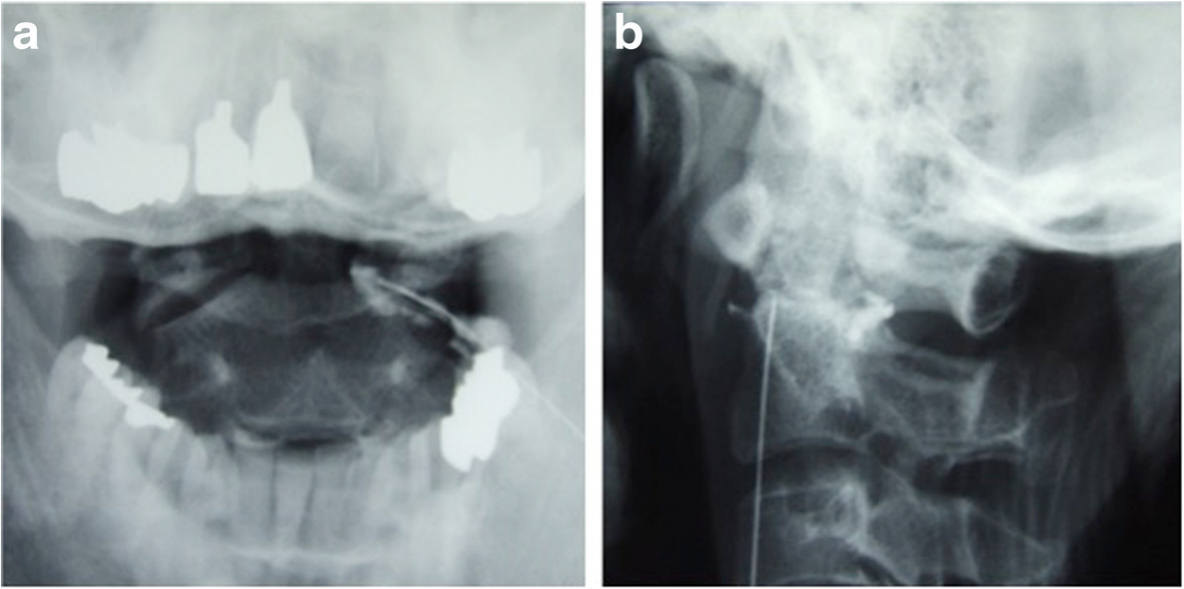
Anterior-posterior (**a**) and lateral (**b**) radiography after radiopaque contrast is injected to lateral atlantoaxial joint. Radiopaque contrast did not go around the dens

Inflammatory disease such as rheumatoid arthritis or crowned dens syndrome (CDS) was considered, and atlantoaxial arthrography was performed. His lateral atlantoaxial joint was punctured under X-ray fluoroscopy. He was placed in a prone position on a fluoroscopic table. Using a block needle, the anterior third of the lateral atlantoaxial joint was punctured. During puncture of the lateral atlantoaxial joint, clear yellow fluid was collected. Radiopaque contrast did not go around the dens (Fig. 4). Cultures later grew methicillin*-*sensitive *Staphylococcus aureus* (MSSA). Histological findings showed no crystals, including calcium pyrophosphate dihydrate. He was finally diagnosed with septic arthritis of the lateral atlantoaxial joint with atlantoaxial subluxation.

After diagnosis, intravenous administration of cefazolin sodium hydrate was begun. Although laboratory data improved 1 week after intravenous administration of antibiotics, his neck pain and stiffness continued. The atlantoaxial region was stabilized with the Brooks procedure [12], including fusion with a bone transplant from his left pelvis, together with wire fixation of the dorsal parts of C1 and C2. Intravenous administration of cefazolin sodium hydrate continued for 3 weeks, followed by oral antibiotics of cefditoren pivoxil for another 3 weeks. His postoperative course was unremarkable. His neck pain decreased and laboratory data normalized 3 weeks after operation.

**Fig. 5:**
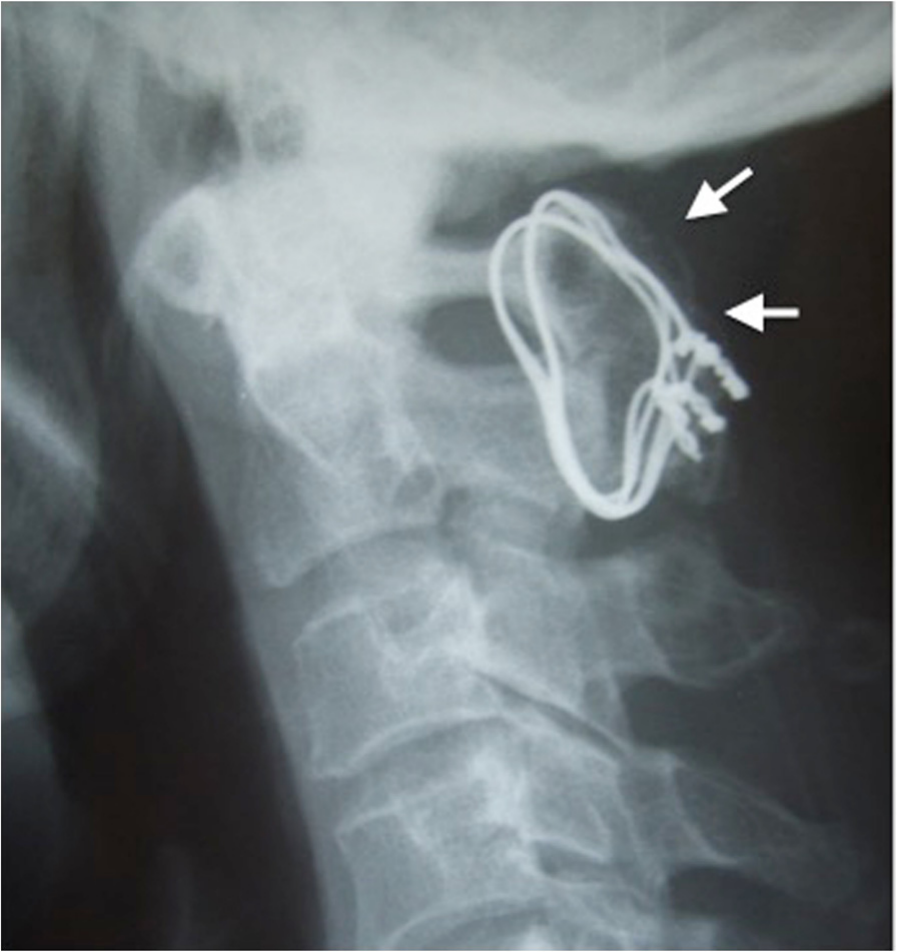
Plain radiography 7 years after operation. Plain lateral radiography shows complete bone union (*arrows*)

Plain radiography showed complete bone union 8 months after the operation. At a follow-up evaluation 7 years after initial onset, he had complete relief of neck pain, and there were no neurological abnormalities. Plain radiography revealed complete bone union (Fig. 5).

## Discussion

Pyogenic infection of the cervical spine has been reported to account for 3 to 20% of all spinal infections [13–16]. Many cases of upper cervical osteomyelitis are associated with osteomyelitis of the odontoid process [17–28]. To the best of our knowledge, only four cases of septic arthritis of the C1–C2 lateral atlantoaxial joint have been reported in the English literature [2–5].

Atlantoaxial subluxation associated with infection at the pharynx and its surrounding tissues is called Grisel’s syndrome [7]. Grisel’s syndrome has also been described in association with postoperative inflammation in surgical conditions such as tonsillectomy and adenoidectomy, in which a clear infective factor is not always proved [8, 9]. The majority of reported cases occurred in patients under 21 years of age [10]; it is rare in adults [11]. The pathogenetic features are still unclear. Decalcification of the vertebra and loosening of the atlantoaxial ligament caused by local infection-related hyperemia are suspected to lead to atlantoaxial subluxation [10]. Septic arthritis of the atlantoaxial joint may cause decalcification of the vertebra and loosening of the atlantoaxial ligament. This is the first report of atlantoaxial subluxation associated with infection at the atlantoaxial joint.

Acute neck pain is often caused by crystal-induced arthritis of the lateral atlantoaxial joint [1] or CDS [29] in the elderly. Vertebral osteomyelitis of the upper cervical spine is very important in the differential diagnosis of crystal-induced arthritis, because early diagnosis is needed for an optimal outcome of vertebral osteomyelitis. Diagnostic delay is an independent risk factor for an unfavorable outcome [6]. In both crystal-induced arthritis and septic arthritis, patients complain of neck stiffness and pain. Patients with crystal-induced arthritis of the lateral atlantoaxial joint typically have a self-limited course, and symptoms usually subside within a few weeks without any aggressive treatment. The time required for the resolution of symptoms in CDS is within 9 days [29]. In septic arthritis, on the other hand, symptoms continue for several months and worsen without appropriate treatment [2–5]. If severe neck pain continues, osteomyelitis of the cervical region should be considered in the differential diagnosis. On CT, bone destruction was seen in the present patient with septic arthritis, while calcification around the dens is seen in most cases of crystal-induced arthritis of the lateral atlantoaxial joint [1]. MRI is the preferred imaging method because of the excellent soft tissue contrast that is achievable. A lateral atlantoaxial joint effusion was visible in the present patient with septic arthritis, but soft tissue swelling or joint effusion is not seen in crystal-induced arthritis of the lateral atlantoaxial joint (Table 1) [1].

**Table 1:** Comparison of crystal-induced arthritis and septic arthritis of the lateral atlantoaxial joint

	Crystal-induced arthritis	Septic arthritis
Neck pain	Severe	Severe
Symptom duration from onset	Resolves within 9 days	Persists for several months without appropriate treatment
Computed tomography	Calcification around the dens	Bone destruction of C1 and/or C2
Magnetic resonance imaging	No marked changes	Joint effusion
Prognosis	Good	Poor without appropriate treatment

Puncture of the lateral atlantoaxial joint is the most effective diagnostic method, although it is thought to be dangerous [30]. In this case, a lateral approach was used, and the fluid collected showed MSSA infection on culture.

Surgery is needed if instability remains [31]. It is possible that this case might have been treated without surgery because cord compression was not very severe. However, stabilization with instrumentation is a safe and effective treatment for pyogenic osteomyelitis [25, 32–34]. In this case, immobilization surgery was chosen because symptoms remained after conservative treatment, and hyperreflexia developed. There are several methods to stabilize the atlantoaxial joint [12, 35–37]. Posterior wiring fixation techniques are not as rigid as posterior atlantoaxial transarticular screw fixation technique or C1-lateral mass screws combined with C2-pedicle screws technique [38]. The use of spinal instrumentation in the infection site has been controversial. In the present case, posterior wiring fixation techniques were considered safe because the wires were placed far from the infected lateral atlantoaxial joint. Posterior atlantoaxial transarticular screw fixation technique or C1-lateral mass screws combined with C2-pedicle screws technique have a risk of penetrating the lateral atlantoaxial joint. This case was successfully treated with posterior wiring fixation techniques and antibiotics. Complete bone fusion was achieved after 8 months, and there was no recurrence for 7 years.

## Conclusions

A patient with septic arthritis of the lateral atlantoaxial joint with subluxation presenting with acute neck pain was successfully treated with antibiotics and fusion surgery. If neck pain continues, septic arthritis of the lateral atlantoaxial joint should be considered, and further examinations are needed.

## Consent

Written, informed consent was obtained from the patient for publication of this case report and accompanying images. A copy of the written consent is available for review by the Editor-in-Chief of this journal.

## Abbreviations

CDS: Crowned dens syndrome
CT: Computed tomography
MRI: Magnetic resonance imaging
MSSA: Methicillin*-*sensitive *Staphylococcus aureus*
